# Operando SXRD study of the structure and growth process of Cu_2_S ultra-thin films

**DOI:** 10.1038/s41598-017-01717-0

**Published:** 2017-05-09

**Authors:** Andrea Giaccherini, Serena Cinotti, Annalisa Guerri, Francesco Carlà, Giordano Montegrossi, Francesco Vizza, Alessandro Lavacchi, Roberto Felici, Francesco Di Benedetto, Massimo Innocenti

**Affiliations:** 1 Department of Chemistry, University of Florence, Via della Lastruccia 3-13, 50019 Sesto, Fiorentino (FI) Italy; 2 INSTM, Research Unit of Florence, Via della Lastruccia 3-13, 50019 Sesto, Fiorentino (FI) Italy; 3 ESRF, 6, Rue Horowitz, F-BP 220, 38043 Grenoble, Cedex France; 4 IGG-CNR, via G. La Pira 4, 50121 Florence, Italy; 5 ICCOM-CNR, Via Madonna del Piano 10, 50019 Sesto, Fiorentino (FI) Italy; 6 SPIN-CNR, Area della Ricerca di Roma 2 - Tor Vergata, Via del Fosso del Cavaliere 100, 00133 Roma, Italy; 7 Department of Earth Sciences, University of Florence, Via La Pira 4, 50121 Firenze, Italy

## Abstract

Electrochemical Atomic Layer Deposition (E-ALD) technique has demonstrated to be a suitable process for growing compound semiconductors, by alternating the under-potential deposition (UPD) of the metallic element with the UPD of the non-metallic element. The cycle can be repeated several times to build up films with sub-micrometric thickness. We show that it is possible to grow, by E-ALD, Cu_2_S ultra-thin films on Ag(111) with high structural quality. They show a well ordered layered crystal structure made on alternating pseudohexagonal layers in lower coordination. As reported in literature for minerals in the Cu-S compositional field, these are based on CuS_3_ triangular groups, with layers occupied by highly mobile Cu ions. This structural model is closely related to the one of the low chalcocite. The domain size of such films is more than 1000 Å in lateral size and extends with a high crystallinity in the vertical growth direction up to more than 10 nm. E-ALD process results in the growth of highly ordered and almost unstrained ultra-thin films. This growth can lead to the design of semiconductors with optimal transport proprieties by an appropriate doping of the intra metallic layer. The present study enables E-ALD as an efficient synthetic route for the growth of semiconducting heterostructures with tailored properties.

## Introduction

Present efforts of materials and surfaces science focus on the development of facile manufacturing techniques which either use processes with a low energy consumption or result in growth with high quality crystalline films. These two aspects of the process are important for semiconducting compounds involved in the development of several new technologies, spanning from nanoelectronics to energy conversion devices. For the last, it is crucial to improve low energy manufacturing techniques in order to increase the energy return of the invested energy, i.e. of the net energy associated with the use of the device in its whole life cycle. On the other hand, high crystallinity and low concentration of defect accumulated during the growth is the main focus in order to opportunely control the electronic and transport properties of the materials. In this context, our work proposes the use of thin layers of sulphides deposited by an electrodeposition technique that allows the deposition of single atomic layers with an accurate control of the crystal structure. Chalcogenide nanomaterials, interesting candidates as new semiconducting nanomaterial due to their tunable band gap and their peculiar transport proprieties^1^, can be grown using several electrochemical methods^2–4^. Among them, the Electrochemical Atomic Layer Deposition (E-ALD) technique is able to build up monolayer-by-monolayer a film, under the bottom up scheme, by alternating the deposition of a monolayer of the metallic element with one of the nonmetallic element, in a cycle which can be repeated several times^5, 6^. Deposition of the monolayers is obtained by means of a surface limited reaction (SLR), i.e. underpotential deposition (UPD). The method is also referred as ECALE (Electrochemical Atomic Layer Epitaxy)^7^ when the deposits can be considered epitaxial. Previous studies^8–10^ were successful in the realization by E-ALD of thin films with binary and ternary composition, as ZnS, CdS, Zn-Cd-S, Cu-S, Cu-Sn-S and Cu-Zn-S, with the aim of growing materials with controlled semiconducting gap properties tuned for photovoltaic applications. While in the case of binary and ternary sulfides including only Cd and/or Zn the obtained phases can be excellently referred to the known structural models^11, 12^, thus allowing a straightforward interpretation of the relative physical and semiconducting properties, the same does not hold true as well in the case of Cu sulfides. It is well known, in fact, that the Cu-S system presents several phases with different stoichiometry and crystal structure^13^ and that the relationships between Cu sulfides and other elements as Zn or Sn evidence only very limited solid solution ranges^14, 15^. Thus, additional structural characterization is needed to improve the understanding of the resulting semiconducting properties^14^. The band gaps of the known structures in the Cu-S system range from 1.2 eV (chalcocite) up to 2 eV (covellite)^16^. Moreover, several structures in the Cu-S compositional field are characterized by a liquid-like ionic conductivity at mild condition. For instance, chalcocite at 100 °C is considered to be a superionic conductor^1^. Thus, these materials are particularly interesting as active material for solar energy conversion. A preliminary *ex-situ* Surface X-ray Diffraction (SXRD) study carried out on Cu-Zn-S and Cu-S thin films, suggested the presence of an epitaxial film grown onto the Ag(111) surface^10^. In the present study, we report on the operando SXRD structural characterization of Cu-S films during their E-ALD growth, with the aim of understanding the growth mechanism and of determining the main structural characteristics of the deposited thin film.

## Phases belonging to the Cu-S system

The portion of the Cu-S system where all natural and synthetic Copper sulfides occur is delimited by two terms, CuS (covellite) and Cu_2_S (chalcocite). Between these two terms, at least 6 different phases have been identified, as stated by Goble in 1985^13^. Evans, and successively other authors, postulated systematic relationships between structure and stoichiometry occurring in the covellite-chalcocite join^13, 17–19^. As a result, Goble fitted the distance between two adjacent sulfur layers along the c-axis versus the Cu/S ratio obtaining a linear relationship^13^. This relation has been used in this work to estimate the stoichiometry of the film.

## Methods

### Beamline and electrochemical setup

The thin films analyzed in the present study were realized starting from analytical reagent grade materials, i.e. Merck CuCl_2_, and Aldrich Na_2_S (as source of Cu and S respectively). HClO_4_ 70% (Merck) and NH_4_OH 33% (Merck) solutions (both having a 50 mM concentration) were used to prepare the pH 9.2 ammonia buffer. The solutions were freshly prepared just before each measurement, using water purified by Milli-Q lab water system.

Commercial Silver single crystal electrodes, sample surface oriented along the (111) plane, were bought from Surface Preparation Laboratory, which guarantees high purity and high angular precision in the chosen orientation. Before each measurement, the electrode surface was cleaned under Ultra High Vacuum (UHV) conditions by performing several cycles of Ar^+^ sputtering at 1 kV followed by annealing at 900 K for about 1 min. The cycles were repeated until a sharp Low Energy Electron Diffraction (LEED) pattern was observed and no contamination was observable by X-ray Photoelectron Spectroscopy (XPS).

The operando SXRD measurements were carried out at the ID03 beamline of the European Synchrotron Radiation Facility (ESRF) in Grenoble using the six circle vertical axis diffractometer installed in the first experimental hutch (EH1)^20^. The electrochemical cell was mounted directly on the diffractometer stage. The employed X-ray energy was 24 keV in order to allow for a high enough transmission through the electrochemical cell (EC) walls and solutions. The electrochemical cell is based on the flow cell concept^21^, which enables for an accurate and stable potential control together with the possibility of a fast exchange of the solutions. The cell is made of PolyEther Ether Ketone polymer, PEEK, a material which couples resistance to the radiation damages, high chemical stability and high X-ray transmittance. A potential drawback of the PEEK choice resides in its polycrystalline nature which results in the presence of intense diffraction rings, which increase the background level and which can hinder some parts of the reciprocal space. To limit this interference, we have reduced the thickness of the EC walls to 100 μm.

The electrochemical cell volume (1.6 mL) was delimited by the working electrode on one side and by the counter electrode on the other side, with the inlet and the outlet for the solutions placed on the side walls of the cylinder. In the three-electrodes scheme, the counter electrode was a glassy carbon stick, and the Ag/AgCl (KCl saturated) reference electrode was placed in the outlet tubing. All potentials reported in this article are quoted with respect to the Ag/AgCl (KCl saturated) reference electrode and connected to a remote controlled potentiostat (Princeton Applied Research PAR263). An automated apparatus consisting of Pyrex solution reservoirs, of solenoid valves and of one distribution valve allows to change the solution present in the cell^21^. The system, fully controlled by the same computer managing the beamline, enables to change the cell solution while avoiding exposure to air of the sample and while still controlling the cell potentials, so preserving its surface from any possible contamination or uncontrolled reaction.

Cu-S films were prepared alternating UPD of the elements synthetizing the compound on the bare Ag (111) substrate, starting with a sulphur UPD followed by a monolayer of copper. Repeating many times this scheme, films with different thickness can be grown. Between two deposition cycles the cell was washed with the buffer solution while always keeping the sample at a controlled potential. For the electrochemical conditions of deposition, i.e. potential and time of deposition, we refer to previous studies^10, 22^.

### Reciprocal space mapping strategy

**Figure 1:**
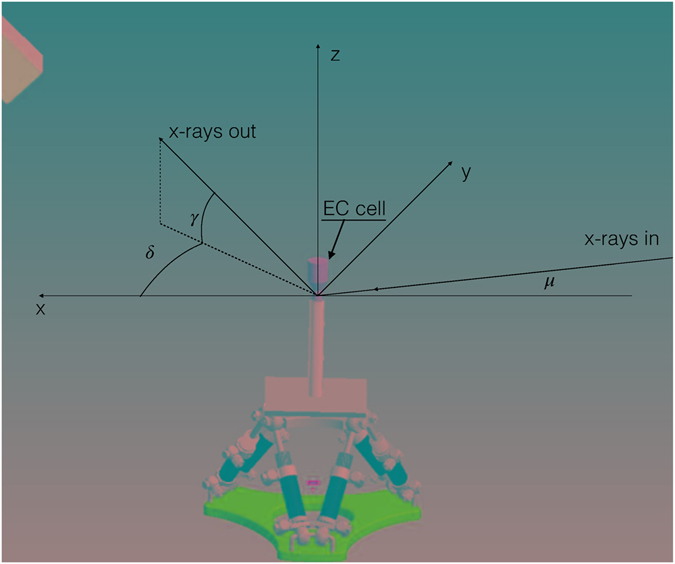
Schematic layout of the SXRD experiment. In SXRD the reference system is referred to the sample surface with the x- and y-axes laying on the sample surface while the z-axis is perpendicular to it. The x axis is along the projection in the x,y plane of the incident X-ray beam. The hexagonal surface cell of the Ag(111) is used as orientation matrix during the experiment. The ***a*** and ***b*** vectors are in the the surface plane while the c vector is parallel to the z-axis. In a SXRD experiment the angle of incidence μ is kept at a constant value (1° in our experiment). Once the orientation matrix of the sample is defined a point of the reciprocal space can be reached by moving the gamma, delta and omega angles. The software takes care of calculating the diffractometer angles for any particular scan in the reciprocal space.

The reciprocal space scans and maps, described in this study, were acquired in the standard SXRD geometry by means of the movements described in Fig. 1.

In this study we report the intensity with respect to a coordinate system referred to the pseudohexagonal surface unit cell of the Ag(111) substrate, in which the surface unit-cell parameters (*a*, *b*, *c*, *α*, *β*, *γ*) are defined so that the **a** and **b** vectors lay on the sample surface along the standard fcc [1 -1 0] and [-1 1 0] directions, while the **c** vector is perpendicular to the surface and parallel to the fcc [111] direction. The amplitude of the three vectors is given by the following relation together with the main surface cell angles as reported by equations (1–3).

1 $$|a|=|b|=\frac{{a}_{0}}{\sqrt{2}}$$

2 $$|c|=\,\sqrt{3}\,{a}_{0}$$

3 $${\rm{\alpha }}={\rm{\beta }}=90^\circ ,\,{\rm{\gamma }}=120^\circ $$

where a_0_ is the lattice parameter of the cubic fcc cell of Ag. In the following, we adopted a reciprocal space metrics where *h*, *k* and *l* are parallel to the a*, b* and c* vectors of the reciprocal surface cell. The most intense peak of the grown Cu_x_S film occurs at (0.73, 0.73, 2.1) in the reciprocal lattice units (rlu) of the reciprocal surface Ag(111) cell defined above. So, the growth of the film can be characterized by performing linear scans in the reciprocal space. We choose two particular reciprocal directions, the first one along the *h* = *k* direction at *l* = 2.1 with *h* = *k* ranging from 0.67 to 0.79 rlu (in-plane scan), and the second one along the *l* direction (out-of-plane scan), normal to the surface, with *l* ranging from 1.8 to 2.4 rlu while *h* = *k* = 0.73 rlu. These scans were performed during the deposition every two Cu/S deposition cycles, and they were used to monitor the appearance of the Bragg reflection and its growth.

Large reciprocal space 2D (h,k) maps were also registered at fixed *l* values (*l* = 1.05, 2.1 and, 3.15 rlu). This procedure was repeated twice during the experiment, at mid-growth, after 30 cycles, and at the end of the growth (60 cycles).

#### Fit of the Bragg peak profiles

A profile analysis of a representative reflection, chosen on the basis of its intensity, was carried out on both the considered crystallographic directions by using a Voigt function^23^. Taking into account that the contributions due to the instrumental resolution and to the monochromaticity of the incident X-ray beam are much smaller than the observed width values^24^ we performed the deconvolution of the Voigt peak’s profile assuming a Gaussian profile for the strain and a Lorentzian profile for the domain size^23, 25–27^. In this theoretical framework, the average crystallite’s strain is directly proportional to the Gaussian width, while the crystallite’s size is inversely proportional to the Lorentzian width. Relative uncertainties of the evaluated parameters (peak intensity, area and widths) were calculated by propagation of the experimental uncertainties of the intensity data. These latter were obtained as Root Mean Square (RMS) of the background. It is worth to notice that the data sets have been projected on the hkl space with a resolution of 0.001 (rlu) and integrated exploiting the Binoculars framework^28^.

#### XRR

X-ray Reflectivity (XRR) curve from a surface does not contain information on the crystalline properties of a film because it depends only on the electronic density profile as a function of the coordinate z perpendicular to the surface. Information, which can be gathered by such measurements, includes the total thickness of the film and its density together with the surface and interface roughnesses^29^. XRR measurements were performed at the end of the growth to investigate the optical thickness of the film. Experimental data have been collected at 60 cycles and projected on the 2 space exploiting the Binoculars software suite^28^ with a resolution of 0.033°. In this context, the XRR data were fitted with GenX^30^ including a roughness factor in the fitting parameter calculated according to the Névot and Croce formalism^29, 31^.

## Results

### Growth of the thin film

**Figure 2:**
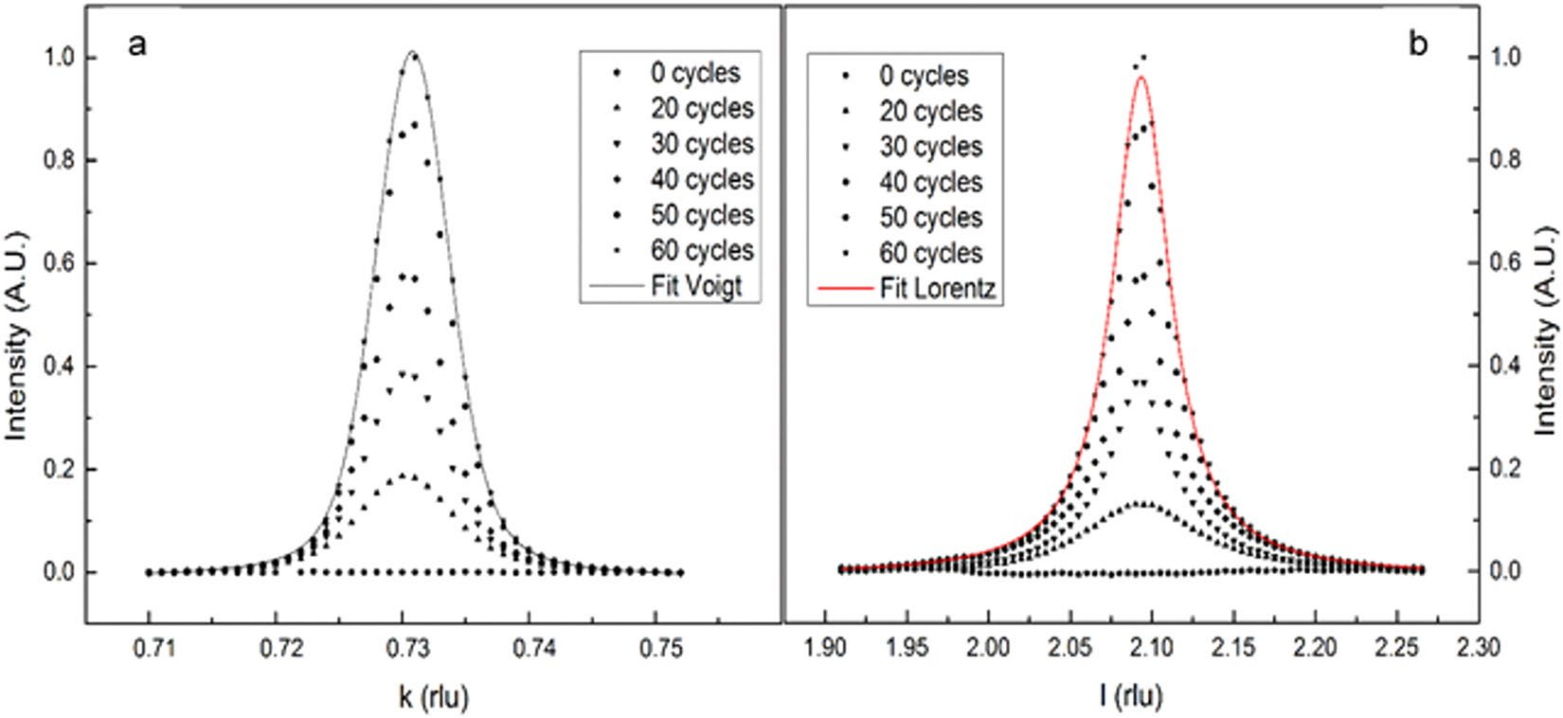
Scans along (h, h, 2.1) (**a**) and along (0.73, 0.73, l) (**b**) at different stages of growth.

Figure 2 shows some of the scans, performed at different stages of the deposition process. In particular Fig. 2a shows scans performed along the *h* = *k* direction at constant *l* = 2.1 rlu while Fig. 2b displays scans along *l*.

The widths and profiles of the (0.73, 0.73, 2.1) Bragg peak along the two different reciprocal directions are very different (Fig. 1). The peak is very sharp along the in-plane *h* = *k* direction (full width at half maximum, FWHM, ~0.02 h *rlu*) and it can be described by the convolution of a constant Gaussian profile with a variable narrow Lorentzian profile (Fig. 1a). This indicates that the film domains are quite large since the initial stages of the growth and that the crystallographic stress due to the lattice mismatch between the film and the Ag(111) substrate is quite small and remain constant during growth.

In contrast, along *l*, the reflection is broader (FWHM ~0.1 *l rlu*) and can be well fitted by using only a Lorentzian function. In this case the peak width is dominated by the finite size of the film, while the contribution due to the crystallite’s strain appears to be negligible (Fig. 1b).

**Figure 3:**
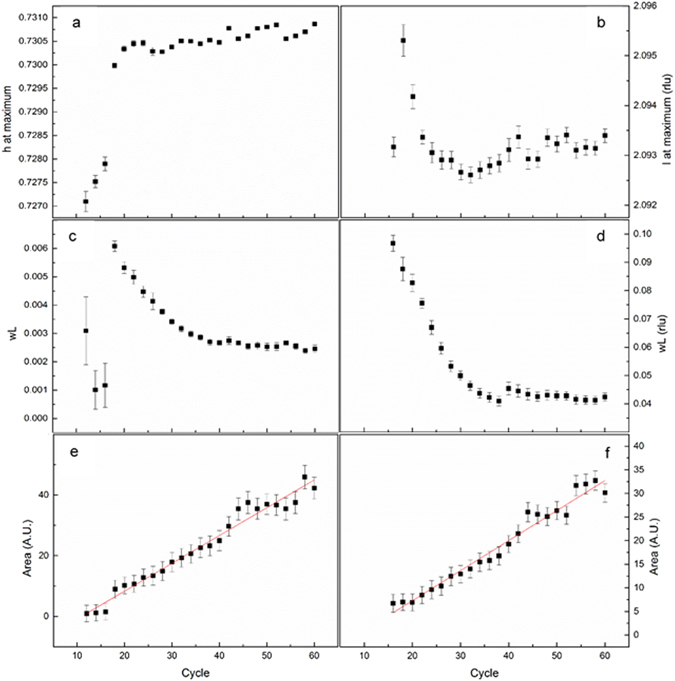
Growth curves for the position at the maximum of the peak, for the Lorentzian breadth (wL) and for the peak area along (h, h, 2.1) (**a,c,e**) and along (0.73, 0.73, l) (**b,d,f**), respectively. The experimental uncertainties are plotted as error bars corresponding to 3 σ.

The SXRD measurements allow us to follow the structural changes which mainly occur up to 40 deposition cycles. The in-plane and out-of-plane scans reported in Fig. 2, provide several information which we summarize in the following (Fig. 3).

**Figure 4:**
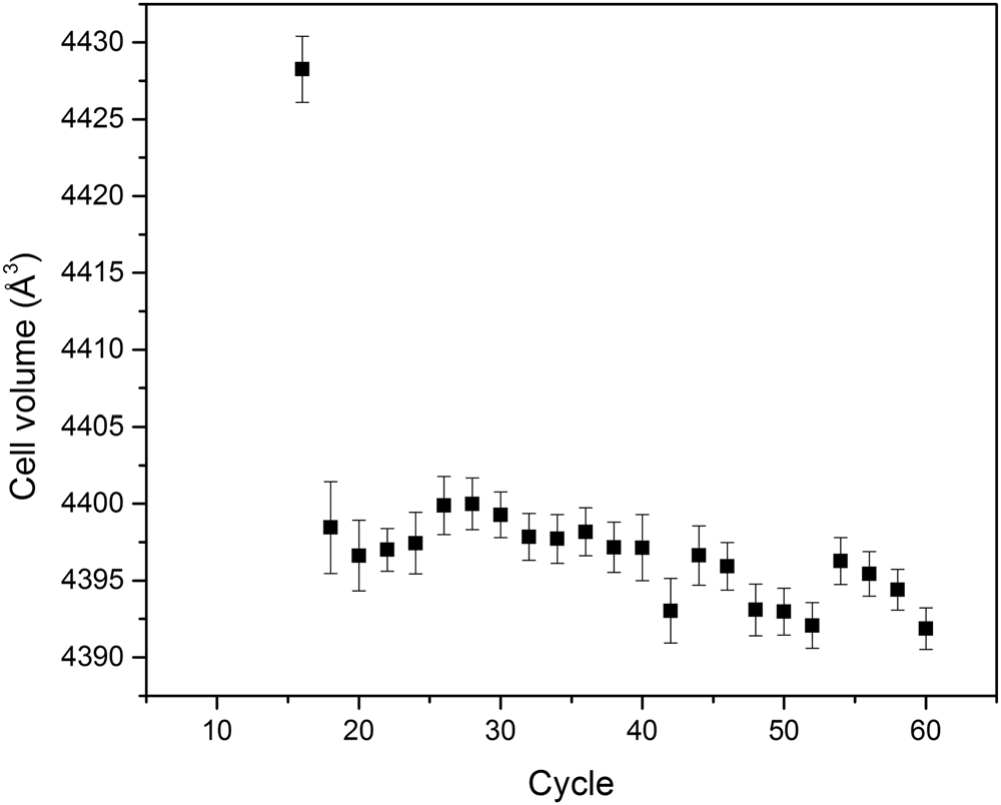
Cell volume in Å^3^ during the growth of the film. The experimental uncertainties are plotted as error bars corresponding to 3 σ.

Figure 2a and b show the position in the reciprocal space of the maximum of the observed peak, which is straightly related to the dimension of the unit cell. Apart from the first cycle at which the peak is observable (i.e. after 16 cycles of deposition), we can deduce that from 20 deposition cycles the crystallographic structure of the film is completely defined. In the early stages of the growth the data seem to indicate that the structure is quite stressed with an expanded in-plane lattice parameter and compressed out-of-plane one, apart for the first point measured after 16 cycles of deposition in which the peak is too broad along the *l* direction to determine its position. The cell volume, reported in Fig. 4, is practically constant along all the observable growth process from 18 deposition cycles onward.

Figures 3c and 2d report the dependence of the Lorentzian widths versus the growth cycles along the in-plane and out-of-plane directions, respectively. The absolute values of the peak width along the in-plane direction suggest that the film nucleates in large islands since the first stages of its growth (Table S1 and Fig. S2 in Supporting Information). During these stages, the domains keep increasing in dimensions until reaching a saturation after about 40 cycles. The lateral dimension of the domains, assessed by the Voigt single line proposed by Vogels^23^, is at least 1000 ± 100 Å, which is a remarkably large value.

In the vertical direction the peak width is much larger, being dominated by the finite size of the film thickness (~140 ± 14 Å, after the 40^th^ deposition cycle). Finite size fringes, characteristic of very well defined thicknesses and of abrupt interfaces, are not visible neither in the l scan crossing the Bragg peak nor in any extended XRR measurement. This is an indication for a quite rough growth, in agreement of the roughness recorded by Bencistà *et al*.^22^. The plots in Fig. 2e and f show that the amount of deposited material goes linearly with the deposition cycles and no saturation is present.

### XRR

**Figure 5:**
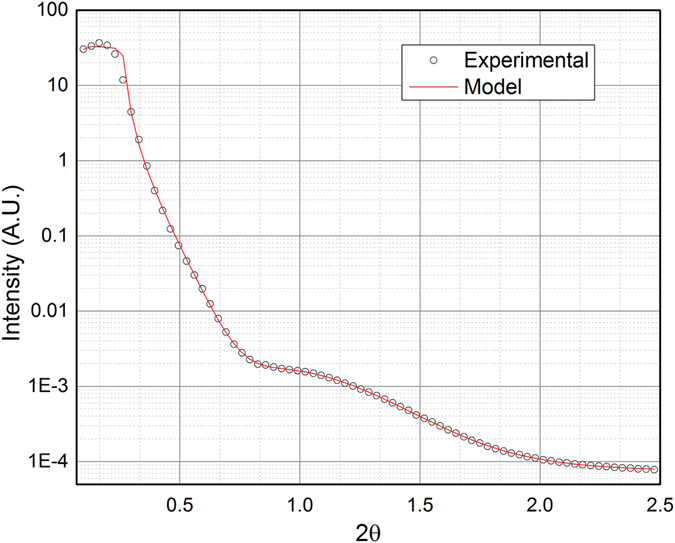
Comparison of the experimental XRR data (open circles) and the model used to fit it (continuous lines).

Experimental data have been fitted with the following stack model: Water/High density layer/Substrate. The results of the best fit is reported in Table 1, together with the estimated uncertainties. Figure 5 shows the XRR experimental data and the related best fits. As previously stated, no fringes are present in the XRR scan impairing the confirmation of the total thickness of the film. However, the fit suggests the formation of a film with an optical density slightly lower than theoretical density (0.0219 A^−3^), high roughness and a very low roughness at the Cu_2_S/Ag(111) interface.

**Table 1:** Result of the fit of the XRR data using the stack model described in paper.

		Model 1
Roughness of the substrate	(Å)	3.25 ± 0.02
Thickness of the bottom layer	(Å)	18,84 ± 0.08
Density of the bottom layer	(Å^3^)	0.0178 ± 0.0001
Roughness of the bottom layer	(Å)	6.54 ± 0.08
Thickness of the top layer	(Å)	—
Density of the top layer	(Å^3^)	—
Roughness of the top layer	(Å)	—

### Reciprocal space maps

In order to gather a better insight into the crystallographic structure of the grown films we measured intensity maps in *hk* planes at different *l* values. The maps show a number of well-defined diffraction peaks. We tried to identify the observed peaks on the basis of the reported structures for the Cu_x_S systems. Particular attention was dedicated to the low chalcocite Cu_2_S structure refined by Evans^17, 18^, which crystallizes in a monoclinic cell (a = 15.246 Å, b = 11.884 Å, c = 13.494 Å, β = 116.35°). The structural model can be described as a hexagonal arrangement of sulphides ions perpendicular to the c axis, with the Cu ions partially filling the triangular sites in the hexagonal plane of sulphide anions, and the triangular sites between two successive planes.

The reason for giving a particular attention to this structure is the presence of strong similarities between the chalcocite structure and the one we observe. First of all, the *l* scans we measured crossing some of the Bragg peaks indicate that the *c* axis periodicity is 6.75 Å. This value is very close to half of the periodicity along *c* observed by Evans^17, 18^ for low chalcocite. This metric is dictated by the distance between two adjacent sulfur layers of 3.37 Å. From this value we can extrapolate that the stoichiometry of our deposited film is Cu_2_S^13^, as in chalcocite.

**Figure 6:**
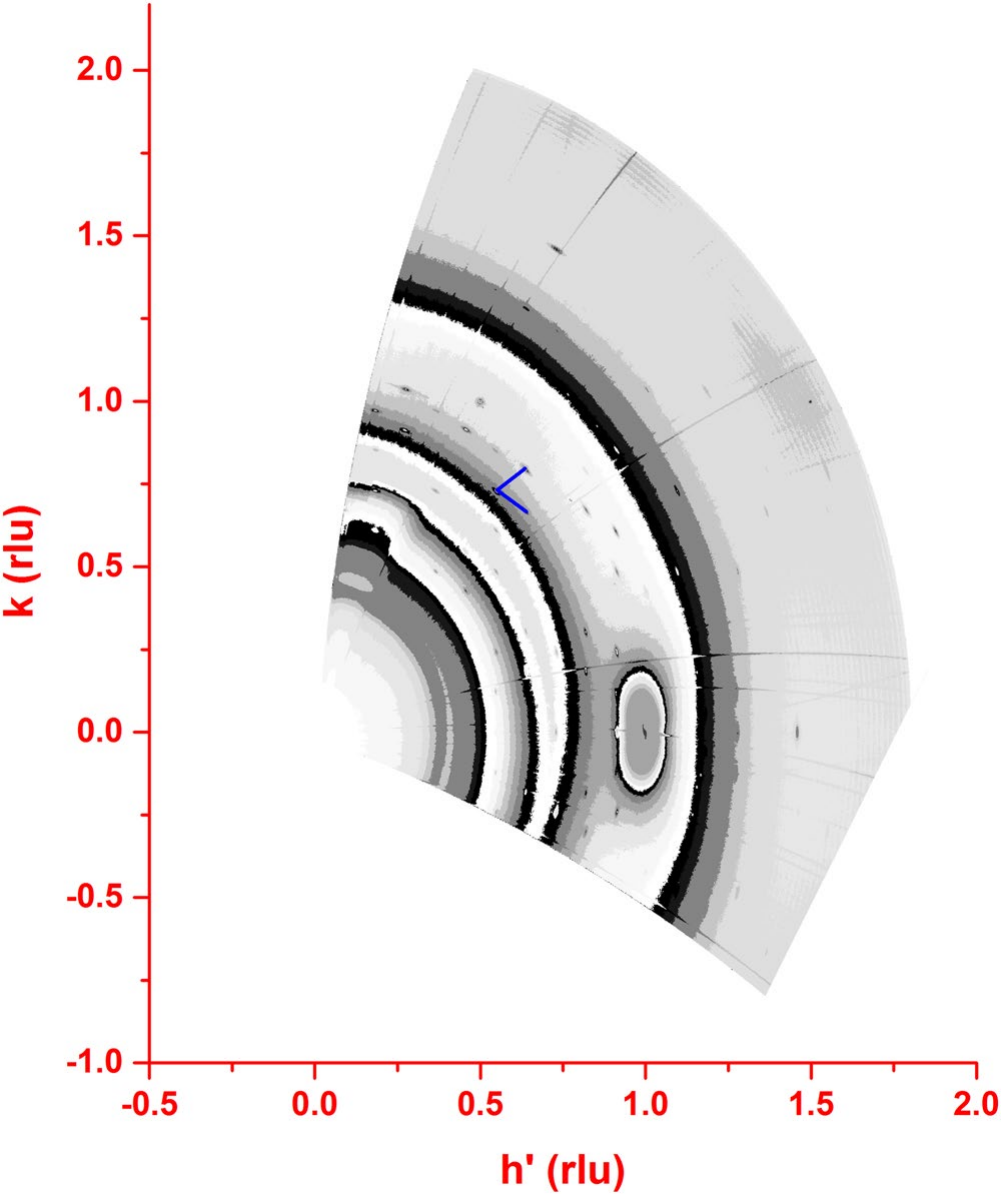
(h, k, 1.05) reciprocal space map of the thin film. Units are expressed in the metrics of the pseudohexagonal silver lattice. The h’ values are related to the h values by the relation: h’ =h + k cos 60°. The blue segments are the unitary vectors of the hexagonal periodicity of the diffraction effects of the film.

The in-plane maps measured at different *l* values, show an apparent pseudohexagonal symmetry (Fig. 5 and Figs S5 and S6 in Supporting Information). The *a* hexagonal cell parameter can be estimated in 27.76 Å, considering the shortest distance between two adjacent Bragg reflections in the map. Interestingly, the unitary vectors of this pseudohexagonal pattern are rotated by 30° with respect of the chosen pseudohexagonal silver frame (Fig. 5). The most intense Bragg reflections, among those registered in our collections occur at (0.73, 0.73, l), (0.73, 0, l), (0, 0.73, l) and at position characterized by doubling the h and/or k values. This suggests that one of the most relevant structural fragments has a 3.96 Å periodicity, which closely corresponds to the S-S distance in the CuS_3_ triangular groups, identified as building units in all the Cu_2-x_S structures. Our data are thus consistent in showing that the Cu_2_S films grown by E-ALD have strong similarities to the chalcocite structural arrangement. However, the (h,k) maps of Fig. 6 definitely point to the occurrence of a new crystal structure.

To confirm this, we projected the pseudo-orthorhombic unit cell, derived from the known structure of low chalcocite, normal to the c* direction. The measured diffraction pattern looks very different from the expected low chalcocite structure even assuming the presence of multiple domains induced by the pseudo 6-fold symmetry of the top Ag(111) surface (Fig. S7 Supporting Information). The present data strongly support the occurrence of a new phase in the Cu_2_S-CuS join, characterized by an in-plane periodicity based on of 7 × 7 triangular CuS_3_ units of the hexagonal-close-packed sulfur framework. A detailed refinement of its structure is prevented because the present set of reflections is not large enough, and because of the interference of the diffuse scattering from the solution (Fig. 5 and Figs S8, S9 and S10 in Supporting Information).

A mention about the structural stability of the films has to be made. The removal of the electric potential, applied to the working electrode, leads to a reversible structural transition, which has not been characterized yet and that will be the subject of further studies (as shown by the Fig. S11 in the Supporting Information).

## Discussion and Conclusions

The main achievement of the present study is the observation of the growth of Cu sulfide over Ag(111) by E-ALD, a film which also exhibits a structural arrangement supporting the idea of a new phase of the Cu-S system, having an apparent pseudohexagonal structural arrangement with unprecedented metrics^32^.

The operando crystallographic structure of the grown Cu_2_S films has strong similarities with the one of the low chalcocite, being based on a hexagonal-close-packed framework of S atoms. In this framework, Cu atoms are partially fixed within the S layers in triangular coordination, and partially occur between adjacent S layers parallel to the surface. This second type of Cu ions is intrinsically characterized by a high mobility in the (a, b) plane^33^. These structural results are in agreement with recent EXAFS studies, revealing a chalcocite-like local structure for copper sulphides in similar systems obtained by means of E-ALD^22^. Considering the orientation of the with respect to the substrate, there is only one non-trivial epitaxial relationship between the substrate and the film (11 × 2) with a strain lower than 5% (−0.8%). Moreover, the structural relationship between monoclinic chalcocite and the high symmetry Cu_2_S structure reported in this study involves the planarization of the CuS_3_ fragments. Such distortion is likely due to a substrate-induced straining which we propose to consider as an epitaxial effect as well. In conclusion, the crystallographic structure suggests an epitaxial process involving linear and angular straining of the in-layer Cu-S bonds, enabling the commensurate growth of chalcocite above the substrate.

The domain size of such films is more than 1000 Å in lateral size and extends with a good structural organization. The high roughness of the film, postulated by the absences of the XRR fringes, hinders the precise analysis of the thickness of the deposited film and it is the marker for inhomogeneous island growth. The asymptotic trend towards a constant value of the width (Fig. 2d), namely the saturation value, in the hypothesis that the width is dominated by the finite size of the thickness and that there is no structural stress, corresponds to a vertical crystallite dimension of about 140 Å, a value which is in good agreement with the number of deposition cycles. The peaks’ intensity increases at every cycle, confirming the continuous growth of film. The experimental evidence that the peak width saturates after about 35 cycles of deposition might also be an indication of the inclusion of structural defects during the growth. However, the crystallite’s size along the perpendicular direction suggests a thickness at least of 140 Å, which seems not compatible with the XRR data. This is likely due to the absence of fringes in the XRR, which disables the separation of the contribution of the optical density and the thickness of the layers. The comparison between the XRR data at 60 cycles and the analysis of the diffraction pattern and profiles suggest the formation of a compact bottom layer and a top layer of Cu_2_S with an optical density lower than the expected. To confirm this hypothesis we run other fittings with two different models:

### Model 1: Water/High density layer/Substrate

#### Model 2: Water/Low density layer/High density layer/Substrate

**Figure 7:**
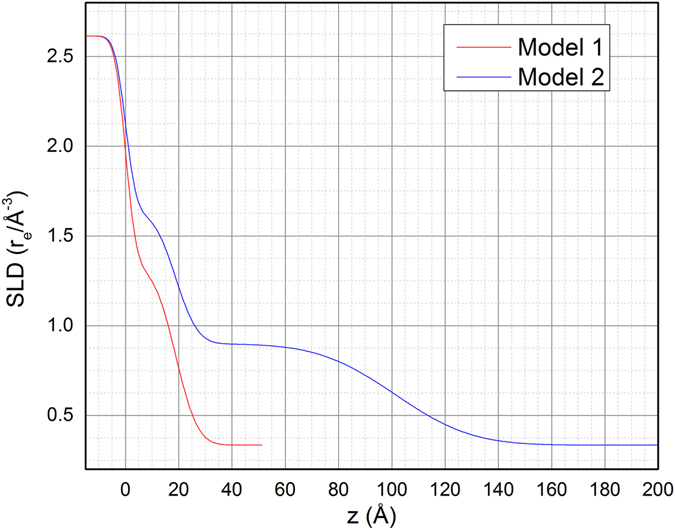
Scattering length densities of the two models fitting the XRR data. A dramatic difference is present for distance higher than 30 Å along z.

For the last model two almost equally good fits have been obtained (Table S3 and Fig. S4 in Supporting information). To confirm the existence of a lower density top layer we compared Model 1 and Model 2 (Table 2) where Model 2 has been fitted with fixed parameters in order to have the same number of estimated parameters. The estimated uncertainties of the two fits in Table 2 show that they can be considered comparably accurate. The relative scattering lengths are reported in Fig. 7, confirming the existence of a lower density top layer. This confirms that the crystallites’ size is compatible with the thickness reckoned by XRR data (see also Model 2 – fit 1 – Table S3 in Supporting Information).

**Table 2:** Comparison between the results of the fitting of the XRR data with the two stack models.

		Model 1	Model 2
Roughness of the substrate	(Å)	3.25 ± 0.02	3.25 ± N/D (Fixed)
Thickness of the bottom layer	(Å)	18,84 ± 0.08	18.84 ± N/D (Fixed)
Density of the bottom layer	(Å^3^)	0.0178 ± 0.0001	0.0178 ± N/D (Fixed)
Roughness of the bottom layer	(Å)	6.54 ± 0.08	6.54 ± N/D (Fixed)
Thickness of the top layer	(Å)	—	82.59 ± 0.06
Density of the top layer	(Å^3^)	—	0.0120 ± 0.0007
Roughness of the top layer	(Å)	—	22.42 ± 0.05

On this basis, the pseudo single crystal pattern showed by the reciprocal space maps, the high optical density of the bottom layer and the low optical density of the top layer suggests the emerging of separated columnar crystallites from a “wetting layer” during the growth. This is compatible with a Stranski-Krastanov growth mechanism. This hypothesis would also be supported by the relevant expected compressive epitaxial relationship. Although the wetting layer is needed to fit the XRR, there are not experimental evidences of the growth of a wetting layer in the analysis of the Bragg peaks. Accordingly, this layer is due to the release of strain occurring before the Bragg peaks become detectable.

**Figure 8:**
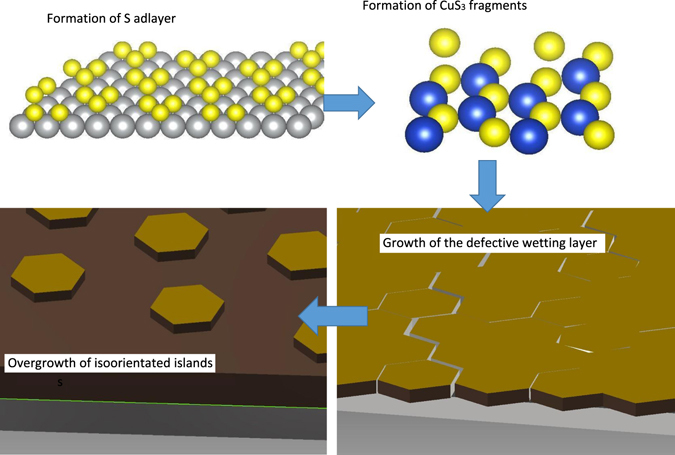
Pictorial representation of the proposed mechanism for the E-ALD growth of Cu_2_S.

From the changes of the profile of the in-plane and out-of-plane Bragg reflections with increasing the cycle number and the SLD at 60 cycles, an approximated description of the growth process can be devised (Fig. 8).

The first S monolayer occurs through the formation of islands of S atoms capping the Ag(111) surface^34^. Then, the first Cu monolayer occurs through the thermodynamically driven formation of Cu-S layer, likely consisting of 2D planar structures based on CuS_3_ units^35^. The first observable stages of the E-ALD process (14^th^−40^th^ cycle) are dominated by the growth of isolated crystals having probably a ratio between the in-plane and out-of-plane dimensions of the order of 10. The individual crystals grow from a wetting layer where grain boundaries could include hexagonal triple junctions. The accumulated strain in the wetting layer is released forming coherent islands which developed the final columnar microcrystalline structure with constant lateral size from the 40^th^ cycle. Such an ordered and strain-free novel structure is expected to have maximized superionic transport properties due to the lack of structural defects, thus opening a research on a new class of semiconductors for applicative purposes. We need to stress that operando SXRD is the only technique able to assess the structure, coherence length and mechanical strain of the crystallite during the growth of the E-ALD ultra-thin films, because of its ability of performing such measurements while maintaining well controlled potential conditions. This is crucial for this kind of systems which show changes of their crystallographic structure as soon as they are in open circuit potential conditions. We can conclude that operando SXRD experiment of the E-ALD process can have a high impact on the design of the growth process for such systems, and their applicability for new photovoltaic applications.

## Electronic supplementary material

Supplementary info

Related Manuscript File
