# Supplementary material for: Operando SXRD study of the structure and growth process of Cu2S ultra-thin films
Source: Sci Rep. 2017 May 9;7:1615. doi: 10.1038/s41598-017-01717-0 (PMC5431668; doi:10.1038/s41598-017-01717-0)
Supplement: Supplementary file 1 — Supplementary info [file 41598_2017_1717_MOESM1_ESM.doc]

**Operando SXRD study of the structure and growth process of Cu_x_S ultra-thin films**

Andrea Giaccherini^1,2,*^, Serena Cinotti^1^, Annalisa Guerri^1^, Francesco Carlà^3^, Giordano Montegrossi^4^, Francesco Vizza^5^, Alessandro Lavacchi^5^, Roberto Felici^6^, Francesco Di Benedetto^2,7,*^ and Massimo Innocenti^1,2,5,*^

1 Department of Chemistry, University of Florence, Via della Lastruccia 3-13, 50019, Sesto Fiorentino (FI), Italy

2 INSTM, Research Unit of Florence, Via della Lastruccia 3-13, 50019, Sesto Fiorentino (FI), Italy

3 ESRF, 6, Rue Horowitz, F-BP 220, 38043, Grenoble, Cedex, France

4 IGG-CNR, via G. La Pira 4, 50121, Italy

5 ICCOM-CNR, Via Madonna del Piano 10, 50019 Sesto Fiorentino (FI) , Italy

6 SPIN-CNR, Area della Ricerca di Roma 2 - Tor Vergata, Via del Fosso del Cavaliere 100, 00133 Roma, Italy

7 Department of Earth Sciences, University of Florence, Via La Pira 4, 50121 Firenze, Italy

* Correspondence to: [andrea.giaccherini@unifi.it](mailto:andrea.giaccherini@unifi.it), francesco.dibenedetto@unifi.it and [m.innocenti@unifi.it](mailto:m.innocenti@unifi.it)

| **Cycles** | **Size along l (Å)** | **Size along h-k (Å)** |
| --- | --- | --- |
| **12** | N/D | N/D |
| **14** | N/D | N/D |
| **16** | 38.5 | N/D |
| **18** | 38.9 | 388.8 |
| **20** | 39.0 | 457.1 |
| **22** | 45.8 | 450.2 |
| **24** | 47.6 | 497.3 |
| **26** | 49.3 | 543.8 |
| **28** | 58.2 | 616.7 |
| **30** | 61.7 | 655.8 |
| **32** | 64.6 | 693.7 |
| **34** | 69.2 | 750.3 |
| **36** | 72.5 | 776.6 |
| **38** | 76.6 | 849.9 |
| **40** | 84.3 | 834.2 |
| **42** | 94.4 | 815.7 |
| **44** | 116 | 832.4 |
| **46** | 116 | 883.2 |
| **48** | 115 | 901.2 |
| **50** | 121 | 917.4 |
| **52** | 117 | 938.8 |
| **54** | 146 | 832.4 |
| **56** | 150 | 883.2 |
| **58** | 153 | 1023 |
| **60** | 141 | 1002 |

Table S1 Crystallographic coherence along c and along the h=k line on the plane during the growth.


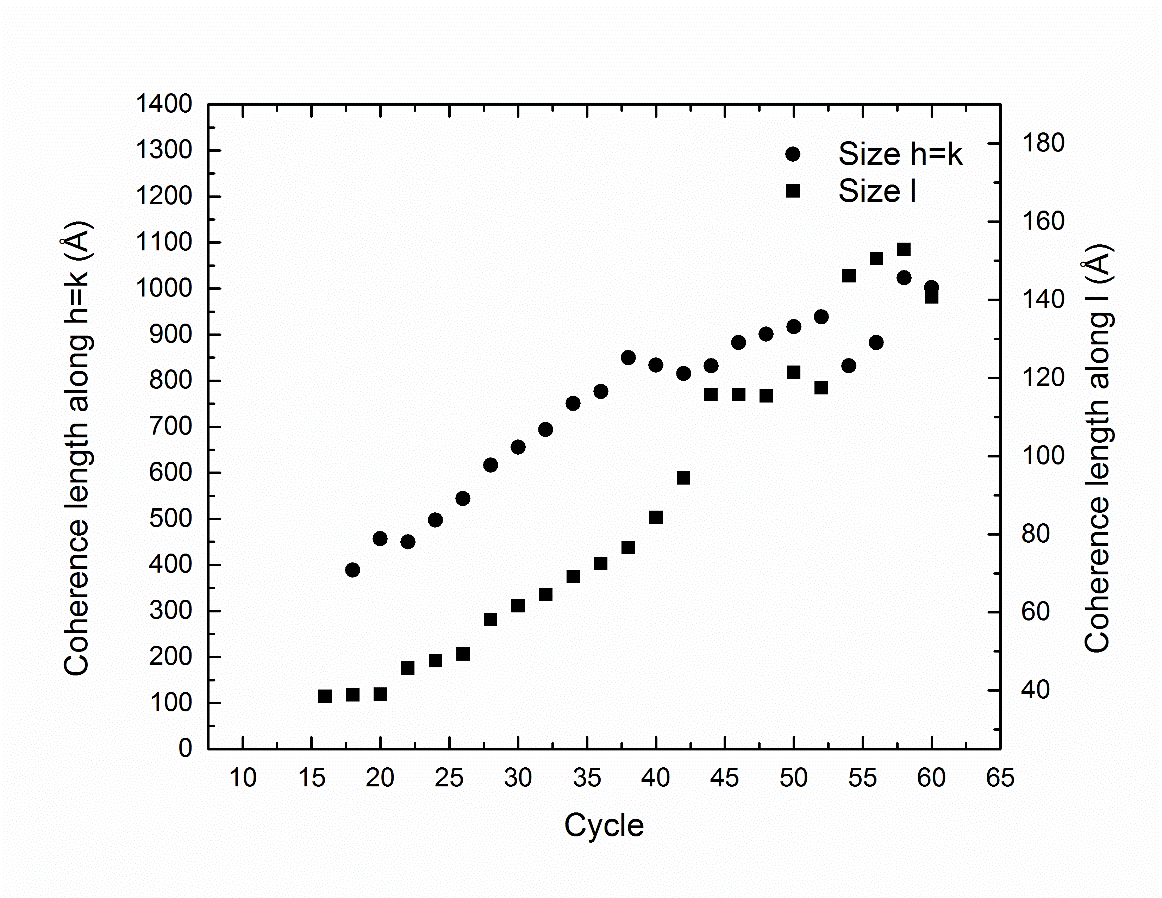


Figure S2 Plot relative to Figure S1.

|  |  | Model1 |  |  | Model 2 |  |  | Model 2 |  |  |
| --- | --- | --- | --- | --- | --- | --- | --- | --- | --- | --- |
| Roughness of the substrate | (Å) | 3.25 | ± | 0.02 | 3.30040 | ± | 0.00001 | 3.29 | ± | 0.02 |
| Thickness of the bottom layer | (Å) | 18.84 | ± | 0.08 | 18.658175 | ± | 0.000006 | 18.75 | ± | 0.04 |
| Density of the bottom layer | (Å^-3^) | 1.78E-2 | ± | 1E-4 | 1.81E-02 | ± | 0.0000004 | 1.829E-2 | ± | 7E-5 |
| Roughness of the bottom layer | (Å) | 6.54 | ± | 0.08 | 6.47707 | ± | 8.E-5 | 6.36 | ± | 0.05 |
| Thickness of the top layer | (Å) | - |  | - | 147.139 | ± | 0.004 | 79 | ± | 1 |
| Density of the top layer | (Å^-3^) | - |  | - | 4.925E-3 | ± | 5.E-6 | 5.8E-03 | ± | 1E-4 |
| Roughness of the top layer | (Å) | - |  | - | 8.48 | ± | 0.01 | 12 | ± | 1 |

Table S3 Three different fits of the same XRR at 60 cycles.


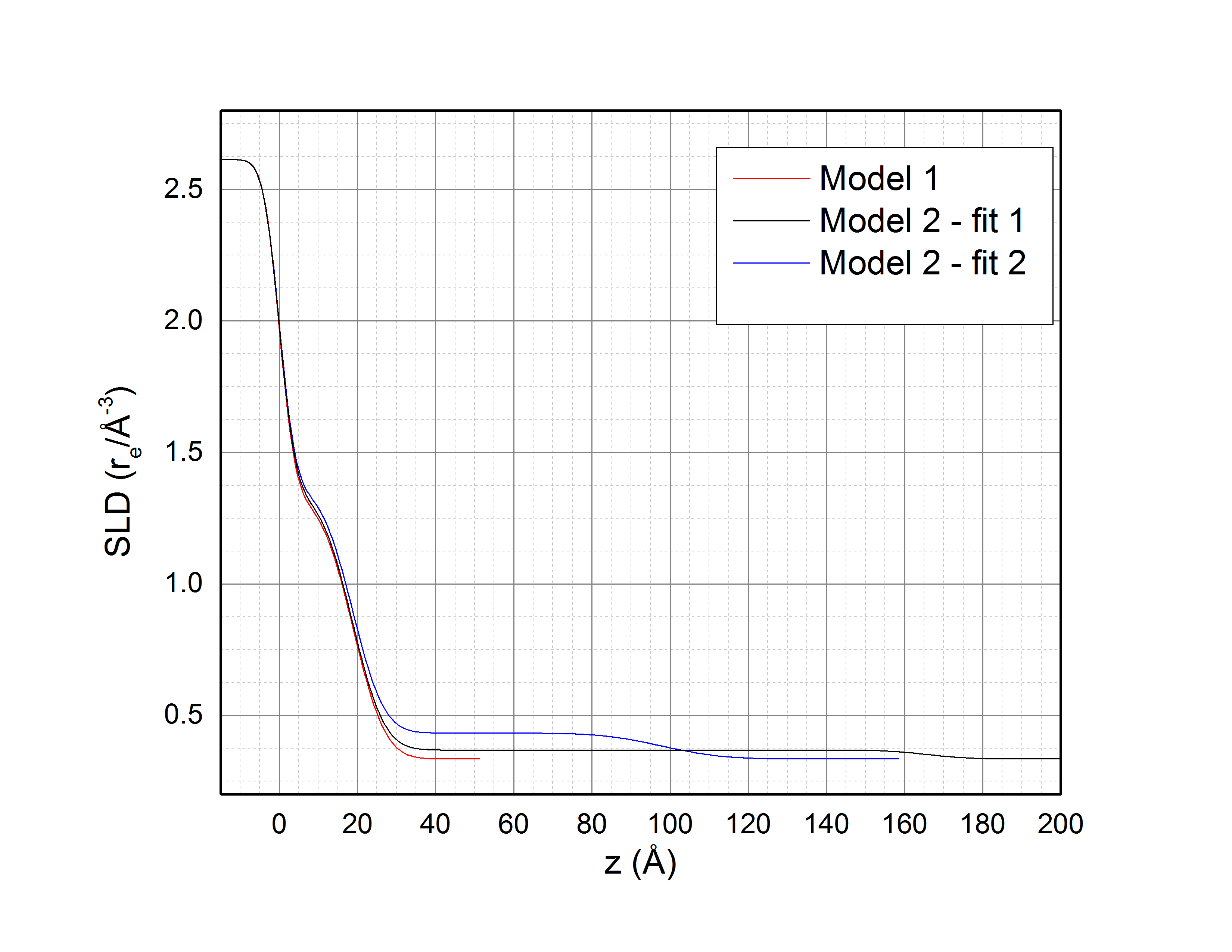

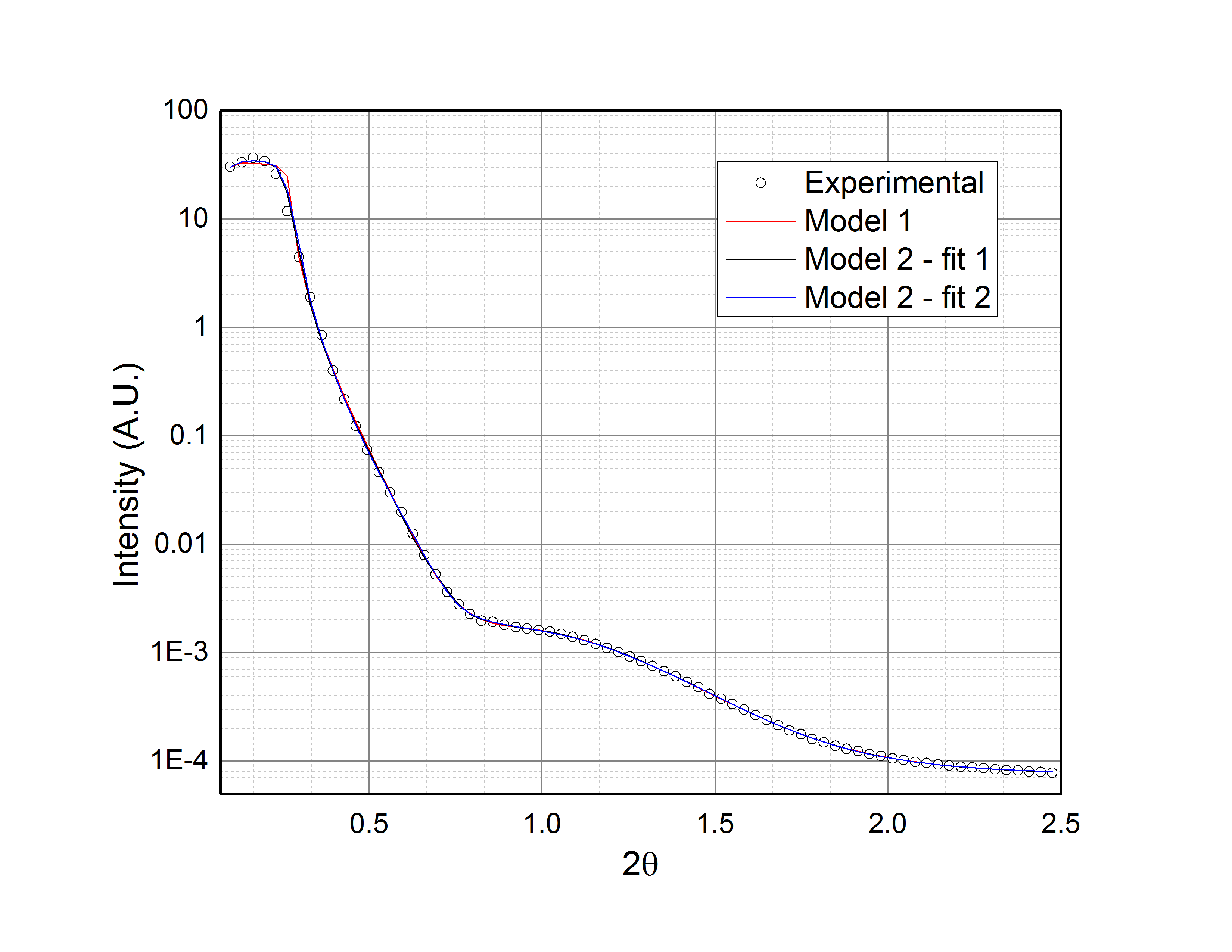


a)

b)

Figure S4 a) plot of the experimental and simulated XRR related to table S3 and b) their SLD.


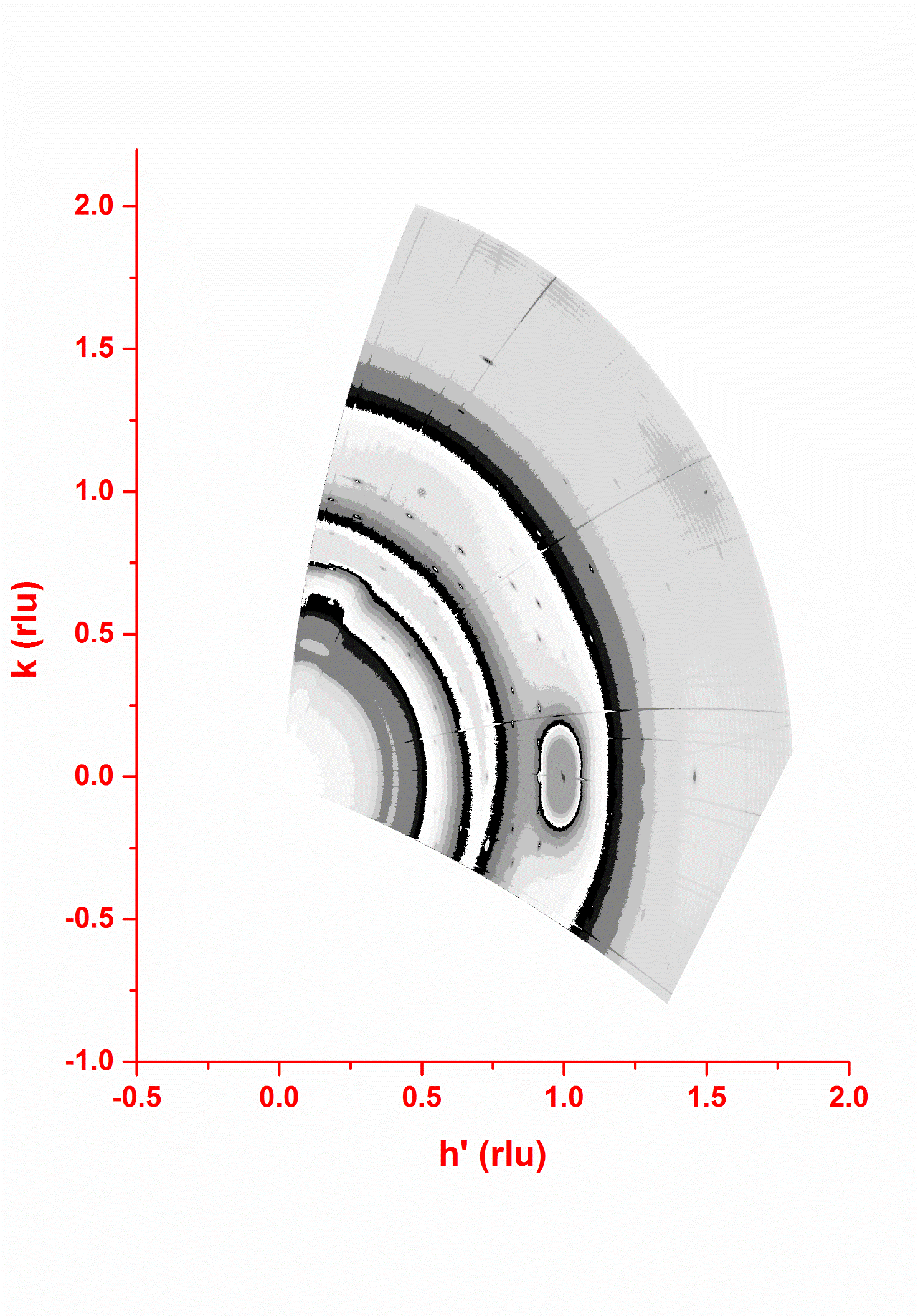


Figure S5 Reciprocal space map at L=1.05 (rlu)


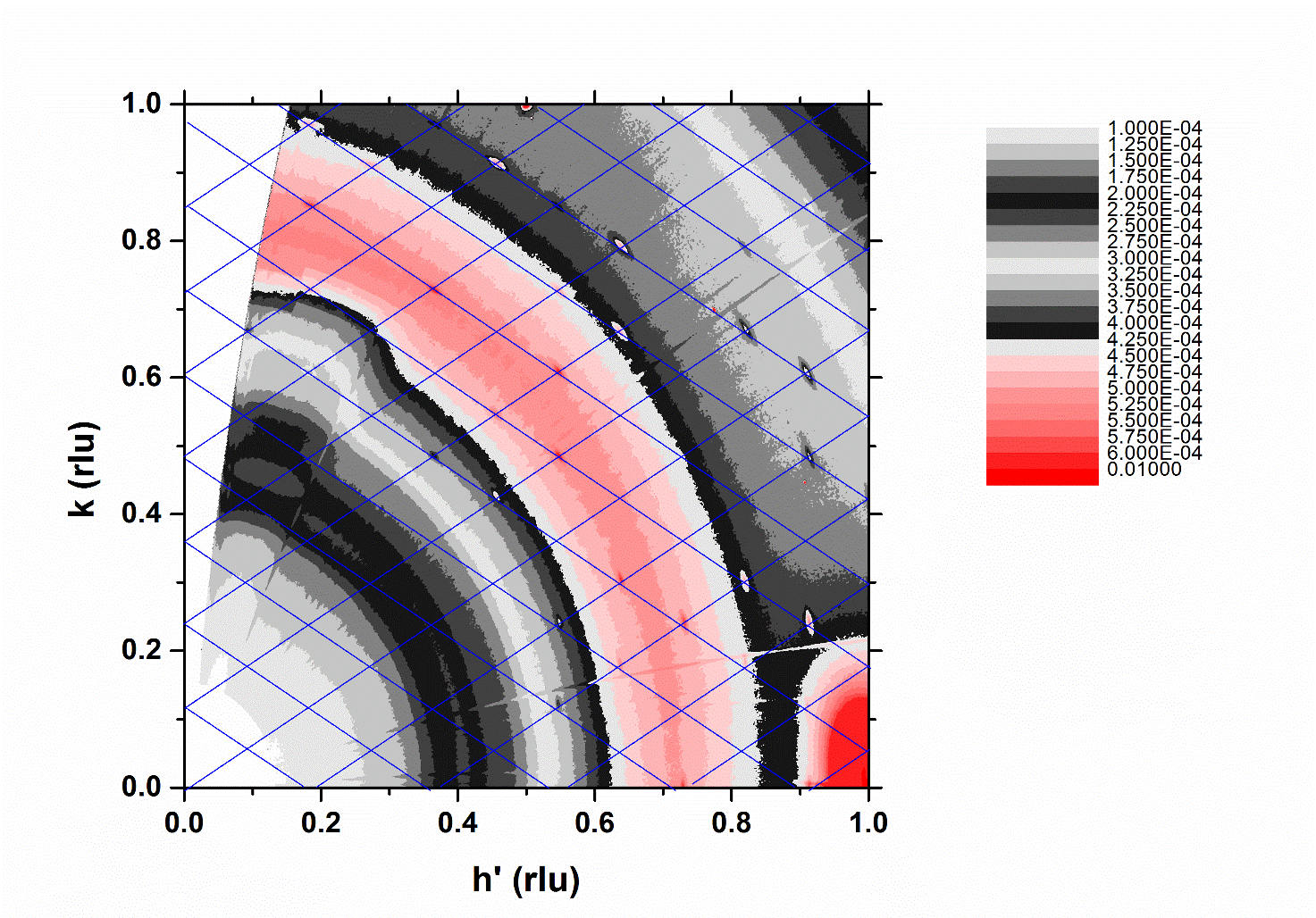


Figure S6 A close-up of the reciprocal space map at l=1.05 (rlu) with the hexagonal mapping of the pattern.


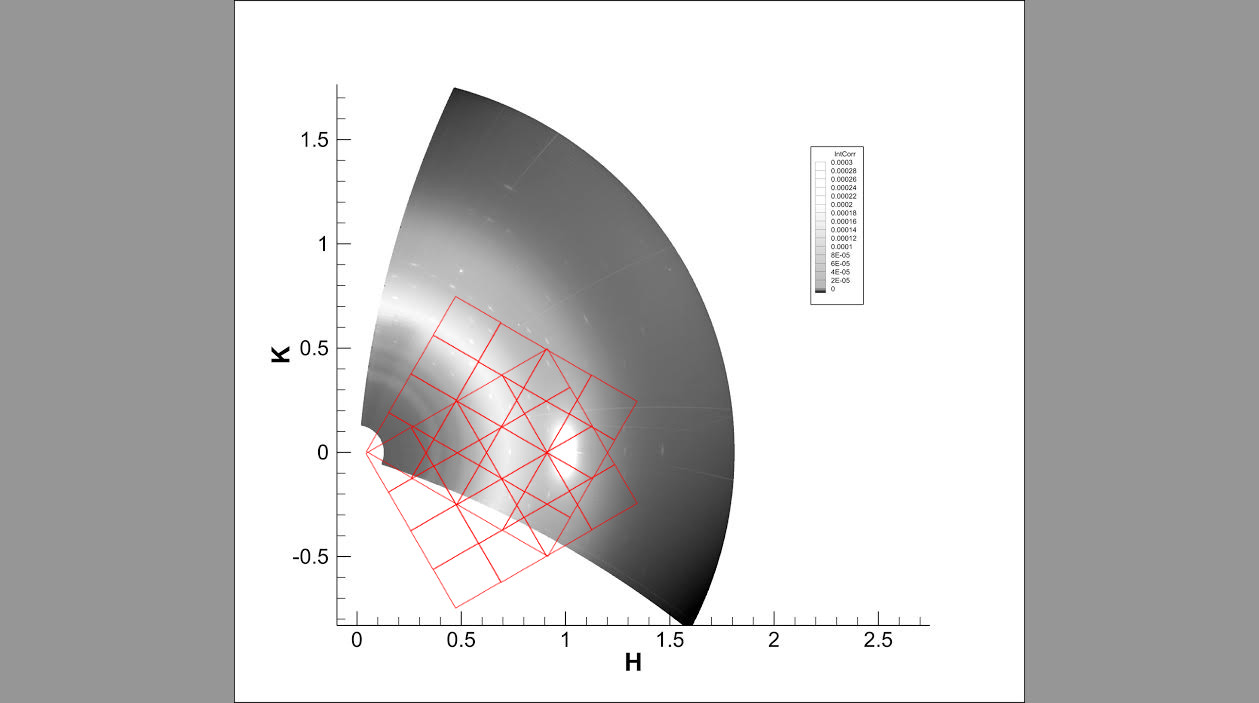

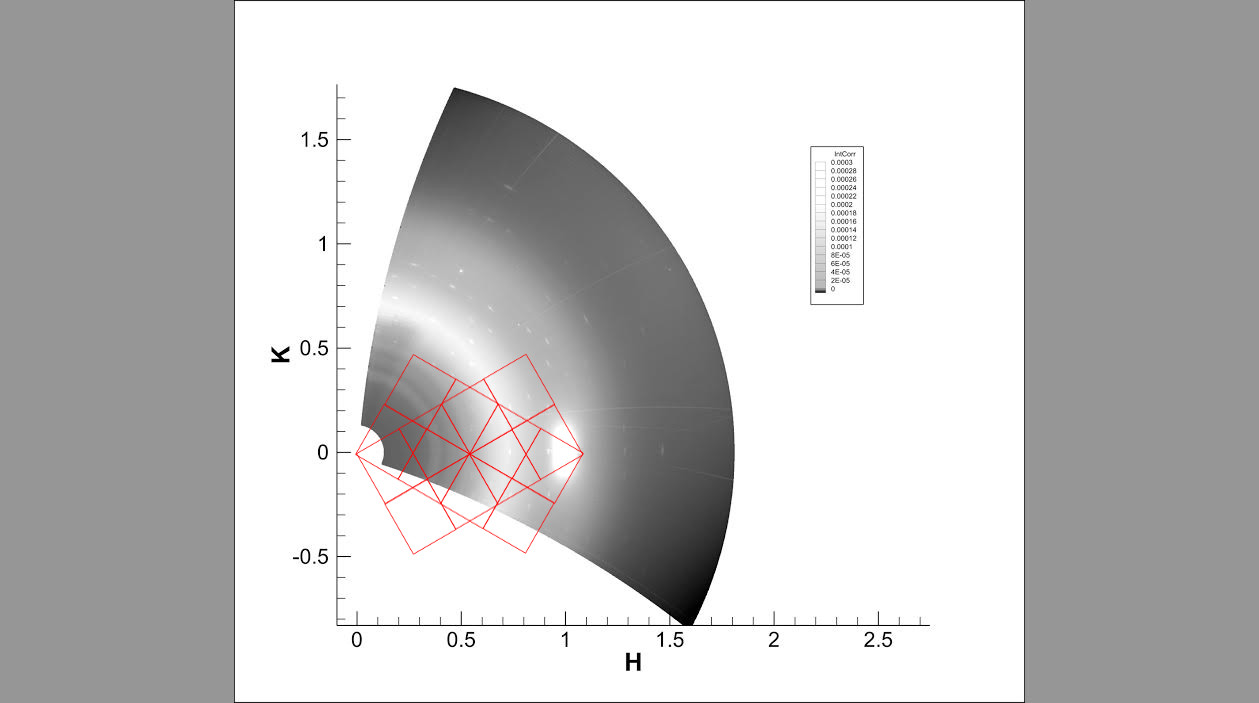


h’

h’

Figure S7 Reciprocal space map at l=1.05 (rlu) with the orthorombic mapping of the pattern based on monoclinic cell of chalcocite (Ref. 17 in the paper).


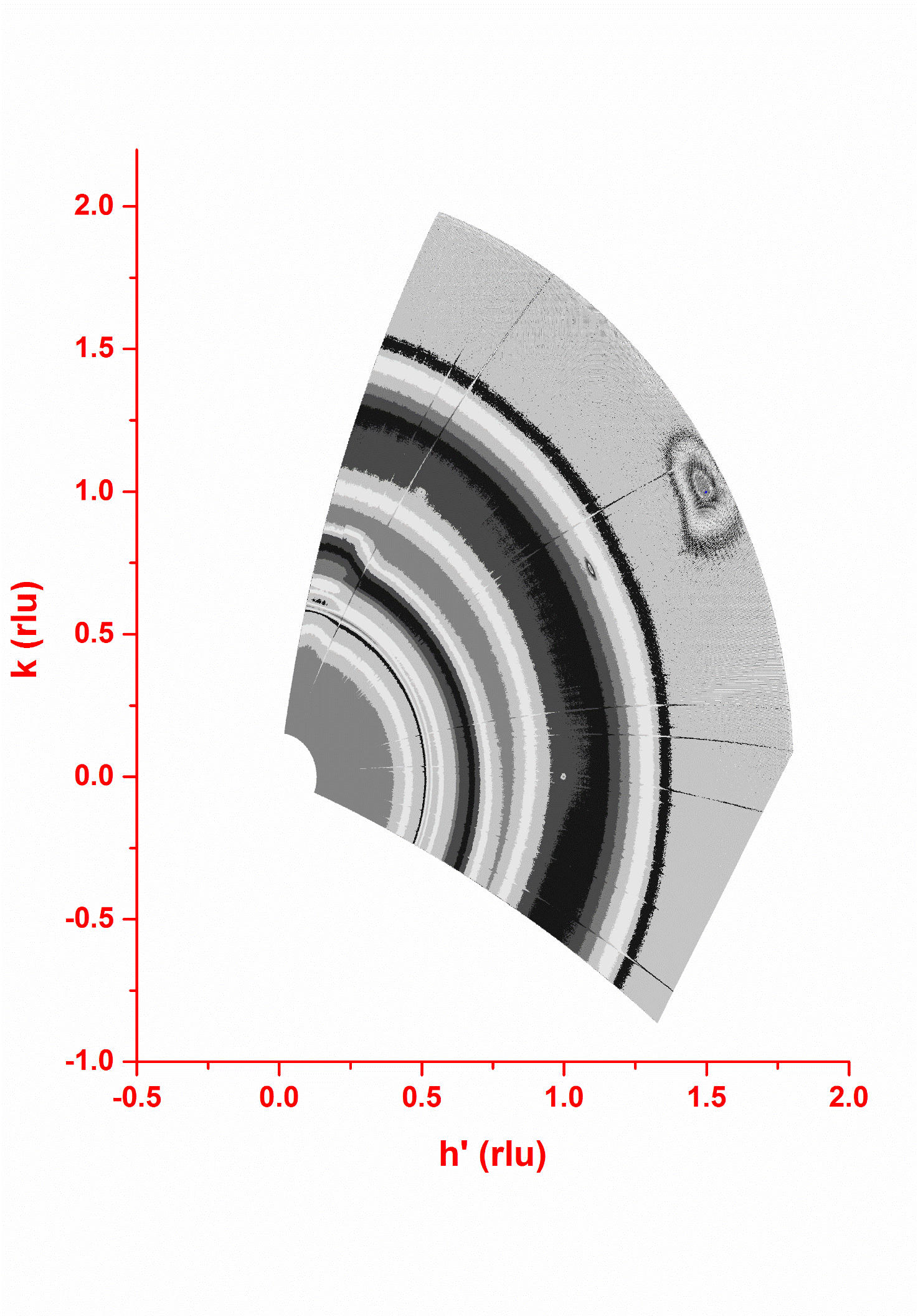


Figure S8 Reciprocal space map at l=0.3 (rlu).


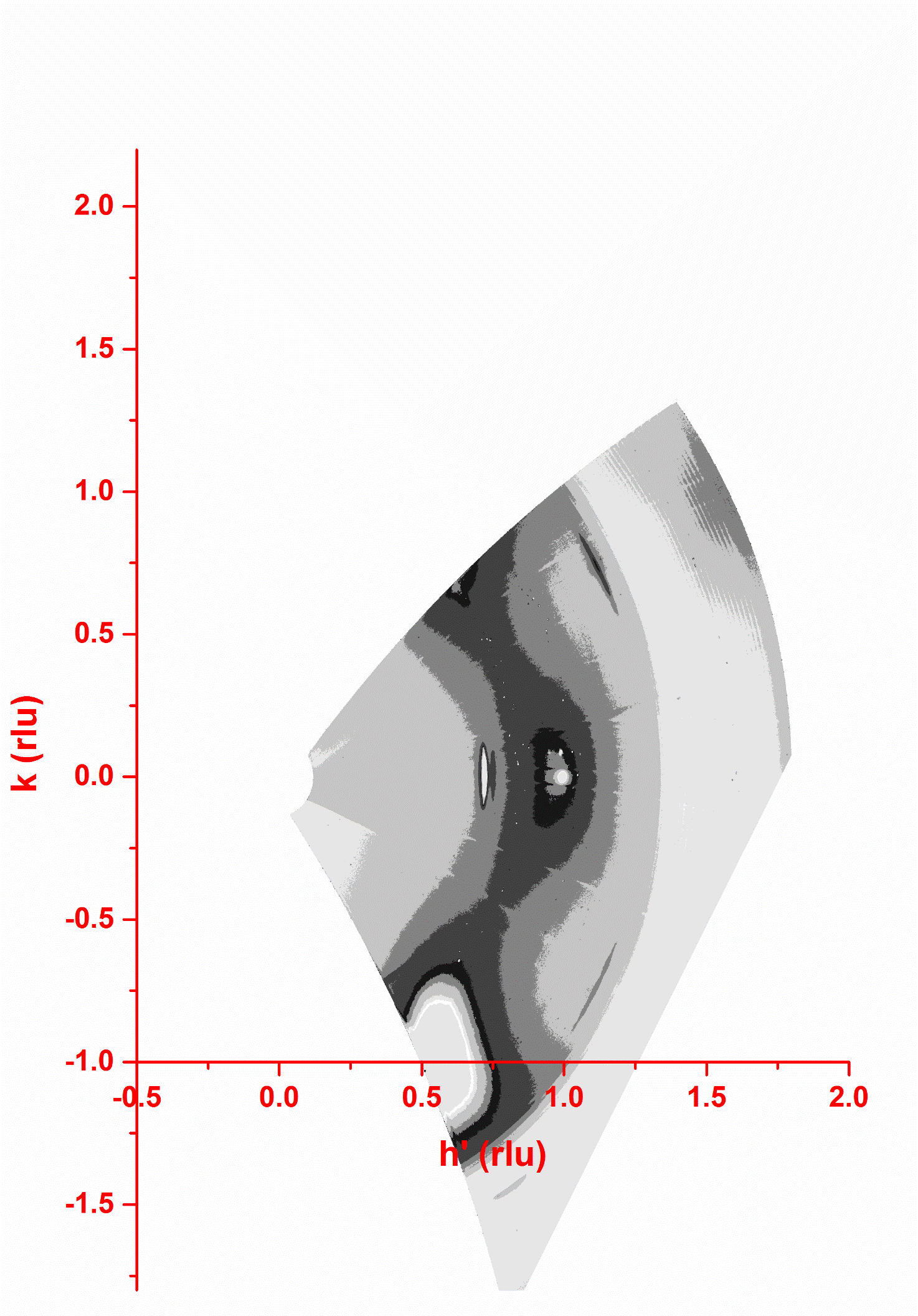


Figure S9 Reciprocal space map at l=2.10 (rlu).


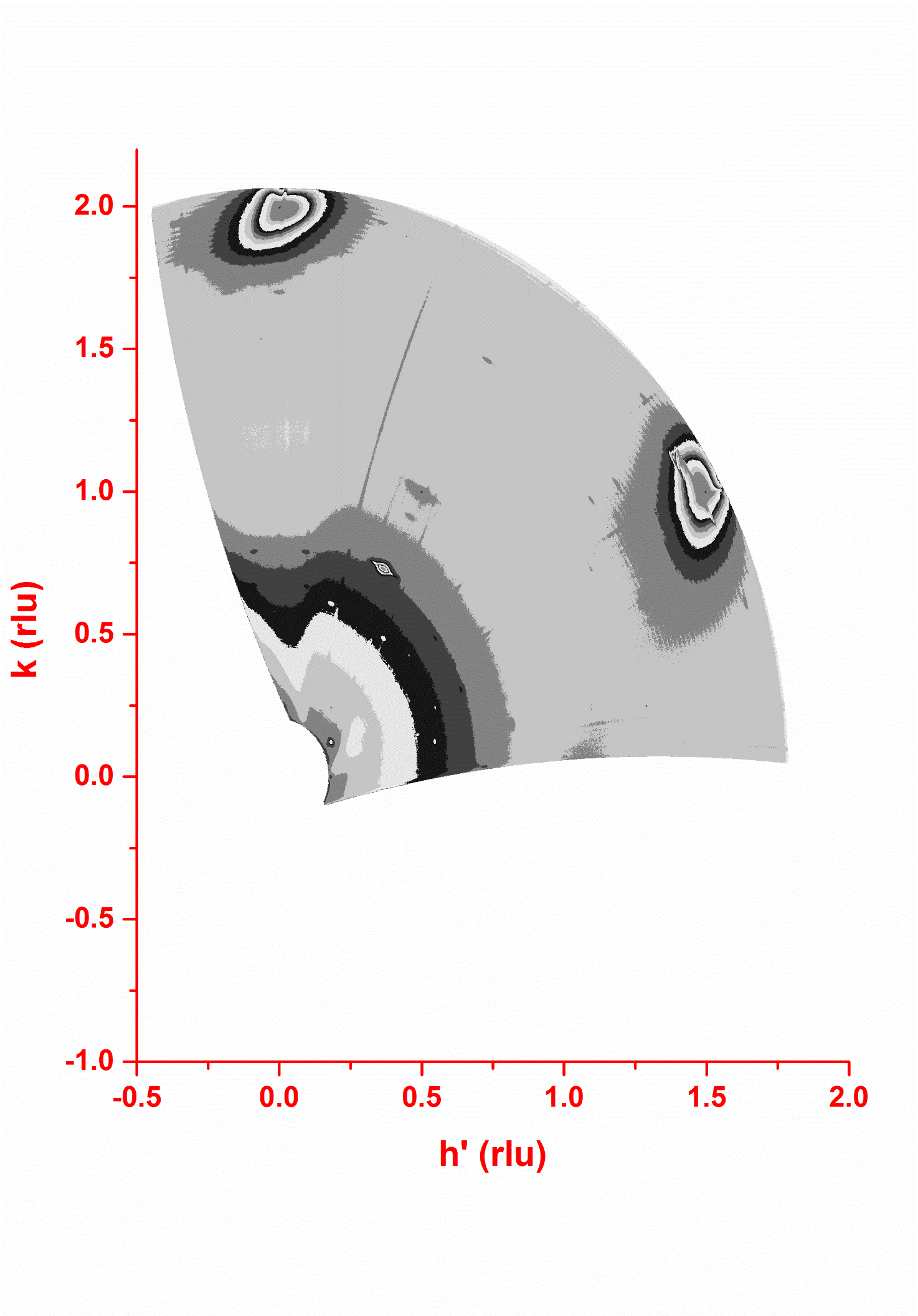


Figure S10 Reciprocal space map at l=3.15 (rlu).


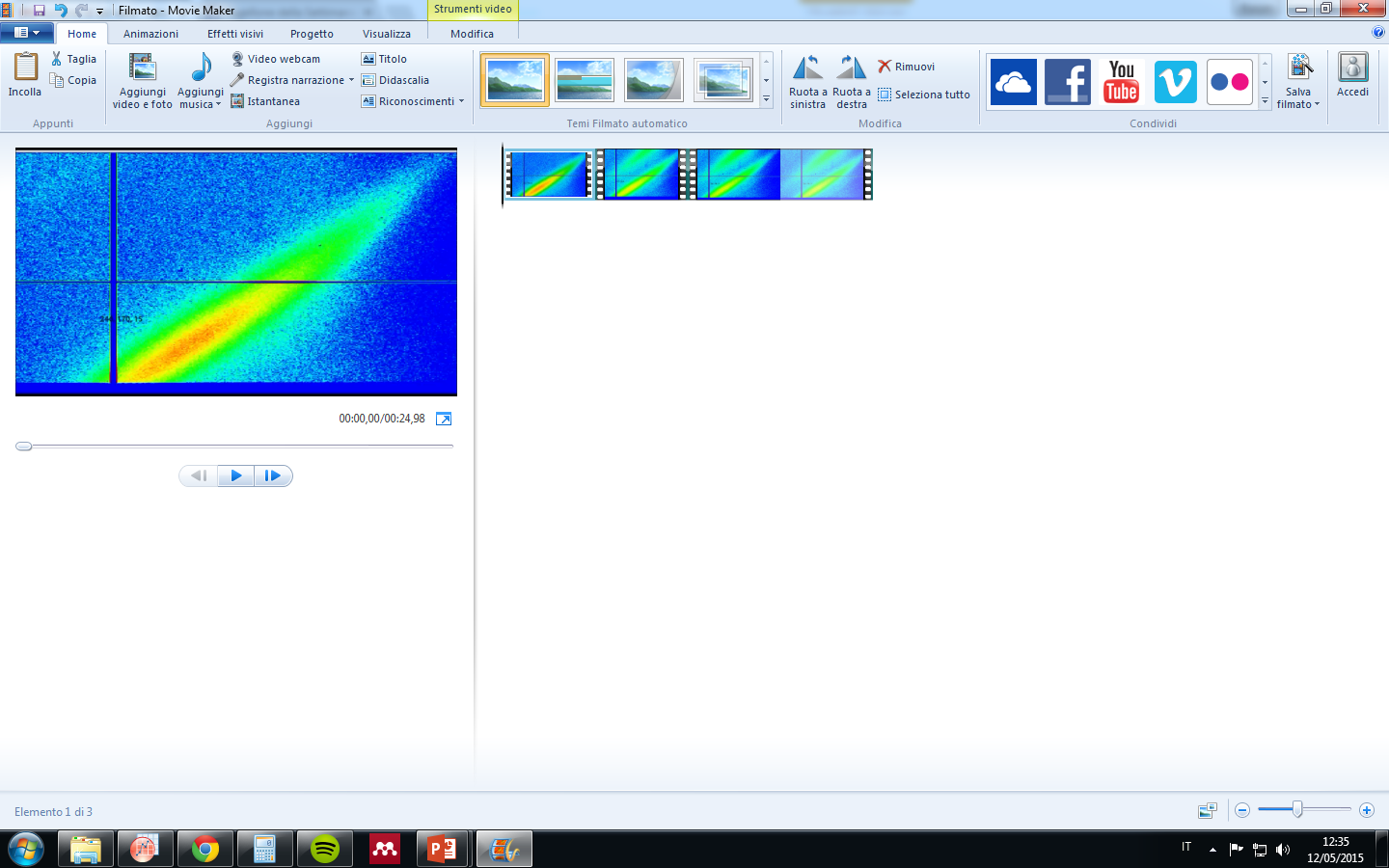
3

a)


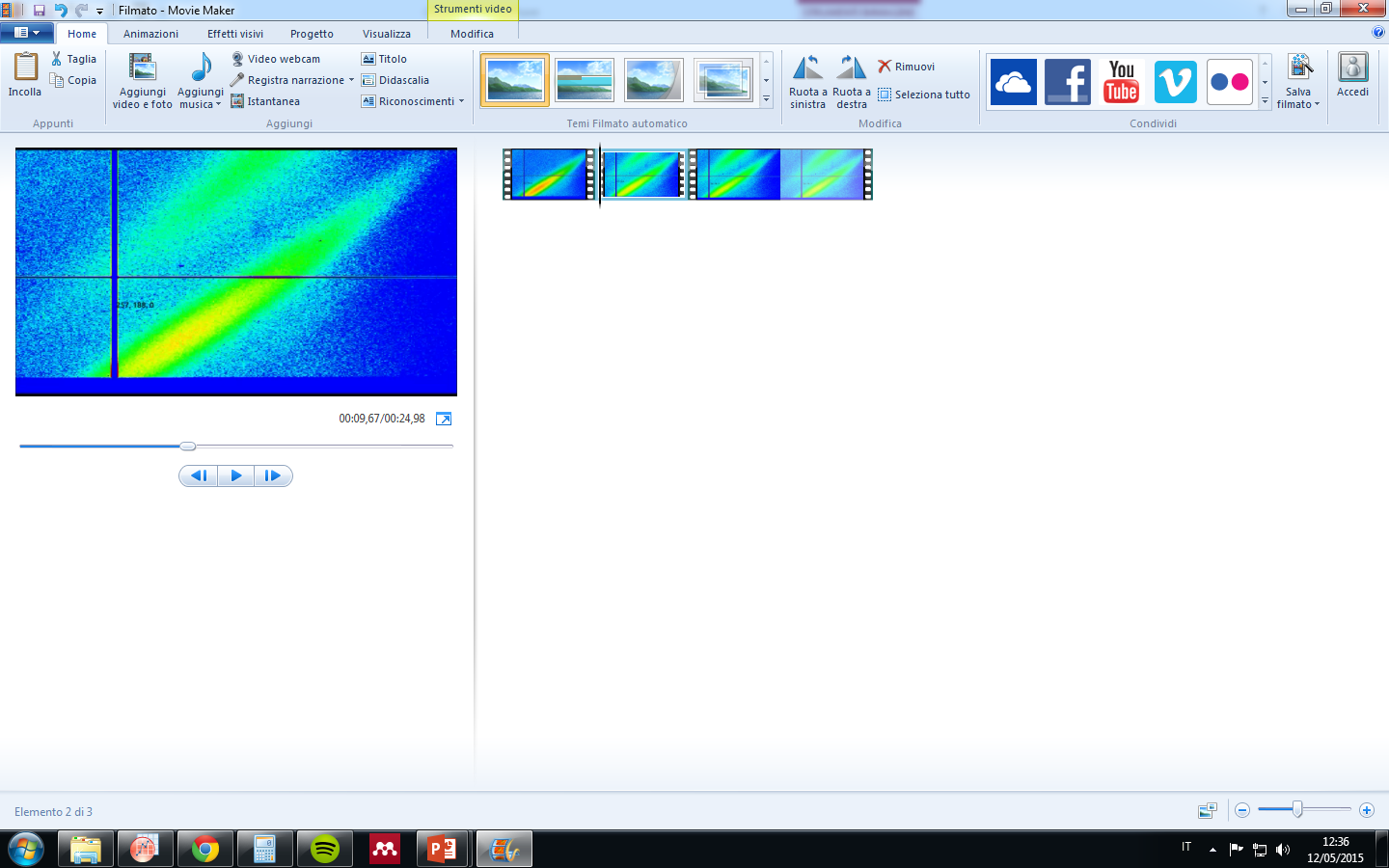


b)


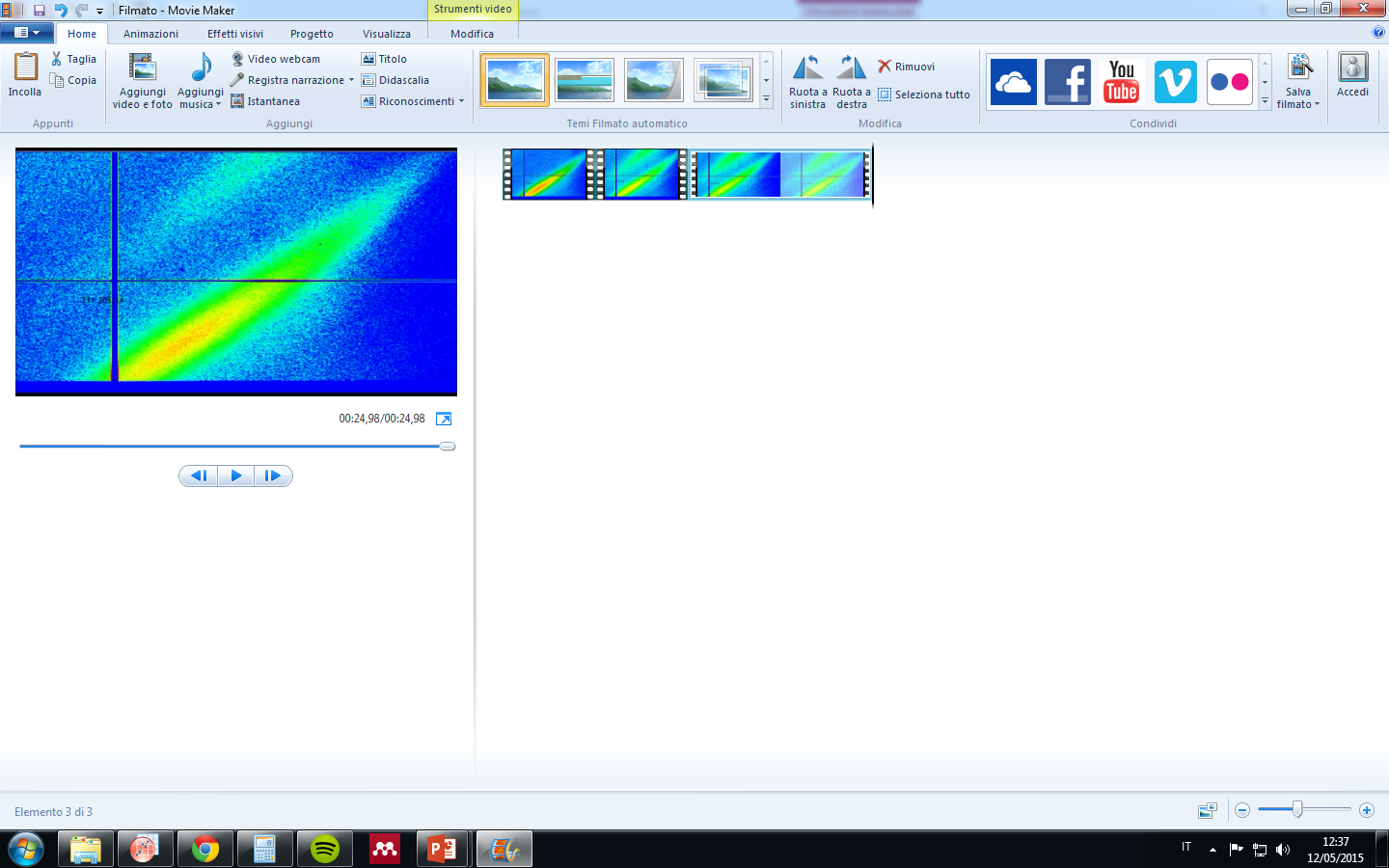


c)

Figure S11 Image registered by the areal detector at (0.73 0.73 2.10) a) just before the removal of the potential, b) showing the emerging Bragg peak after about 10 seconds and c) about 10 seconds after the re-application of the potential. See also video S12.

Video S12 Video registered by the areal detector at (0.73 0.73 2.10) during the evolution of the structure after the removal of the potential and after the re-application of the starting potential (-0.68V).
